# Cleft Palate Is Caused by CNS Dysfunction in *Gad1* and *Viaat* Knockout Mice

**DOI:** 10.1371/journal.pone.0009758

**Published:** 2010-03-19

**Authors:** Won-Jong Oh, Joby J. Westmoreland, Ryan Summers, Brian G. Condie

**Affiliations:** 1 Developmental Biology Group, Department of Genetics, University of Georgia, Athens, Georgia, United States of America; 2 Institute of Molecular Medicine, Medical College of Georgia, Augusta, Georgia, United States of America; Ecole Normale Supérieure de Lyon, France

## Abstract

**Background:**

Previous studies have shown that disruption of GABA signaling in mice via mutations in the *Gad1*, *Gabrb3* or *Viaat* genes leads to the development of non-neural developmental defects such as cleft palate. Studies of the *Gabrb3* and *Gad1* mutant mice have suggested that GABA function could be required either in the central nervous system or in the palate itself for normal palatogenesis.

**Methodology/Principal Findings:**

To further examine the role of GABA signaling in palatogenesis we used three independent experimental approaches to test whether *Gad1* or *Viaat* function is required in the fetal CNS for normal palate development. We used oral explant cultures to demonstrate that the *Gad1* and *Viaat* mutant palates were able to undergo palatogenesis in culture, suggesting that there is no defect in the palate tissue itself in these mice. In a second series of experiments we found that the GABA_A_ receptor agonist muscimol could rescue the cleft palate phenotype in *Gad1* and *Viaat* mutant embryos. This suggested that normal multimeric GABA_A_ receptors in the CNS were necessary for normal palatogenesis. In addition, we showed that CNS-specific inactivation of *Gad1* was sufficient to disrupt palate development.

**Conclusions/Significance:**

Our results are consistent with a role for *Gad1* and *Viaat* in the central nervous system for normal development of the palate. We suggest that the alterations in GABA signaling lead to non-neural defects such as cleft palate as a secondary effect due to alterations in or elimination of fetal movements.

## Introduction

The mechanism leading to non-neural developmental defects in mice homozygous for null alleles of the genes *Gad1*, *Gabrb3* or *Viaat* (*Slc32a1*) has been an enigma [1], [2], [3], [4], [5]. Each of these genes encodes a component required for GABA neurotransmission including GABA synthesis (*Gad1*), GABA vesicular transport (*Viaat*) and the postsynaptic response to GABA (*Gabrb3*). The most prominent phenotype noted by us and other investigators has been the surprising presence of a cleft palate in neonatal mice homozygous for null mutations in *Gad1*, *Viaat* and *Gabrb3* [3], [4], [5], [6]. In addition, possible associations between human oral clefts and alleles of *GAD1* or *GABRB3* have been reported [7], [8], [9]. Overall, this literature suggests that understanding the developmental mechanisms that lead to oral clefts in the *Gad1*, *Viaat* and *Gabrb3* mice might lead to new insights into the origin of human oral clefts, a common congenital disorder.

Several studies have been performed to determine whether GABA signaling is required in the CNS or in peripheral tissues for normal palate formation in *Gad1* and *Gabrb3* mutant mice. These studies have drawn different conclusions depending on the gene examined and the experimental approach taken. In the case of *Gabrb3*, two studies support a role in non-neural cell types for normal palatogenesis. In one case, transgenic expression of *Gabrb3* from a neuron-specific enolase promoter failed to complement the cleft plate phenotype of a *Gabrb3* null mutation, suggesting that non-neural expression of *Gabrb3* was required for normal palate formation [10]. Another study showed that palate development was normal in a pan-neuronal-specific knockout of the *Gabrb3* gene, again suggesting that *Gabrb3* function was required in the palate or other craniofacial structures for normal palatogenesis [11]. In the case of *Gad1*, previous studies indicated that the cleft palate phenotype of *Gad1*-/- pups was due to a lack of fetal oral movements as well as inhibition of palate shelf elevation due to the abnormal position of the tongue between the shelves [12], [13]. This analysis of the *Gad1* phenotype was consistent with a requirement for GABA signaling in the CNS for normal fetal movement that in turn allows normal palate development. However, several papers have reported GABA, *Gabrb3* or *Gad1* expression in developing craniofacial structures such as the palate, oral epithelium, tooth placodes or condensations, tooth buds and palate medial edge epithelium [4], [10], [14], [15], [16], [17]. The presence of *Gad1* mRNA or protein as well as GABA in these structures is consistent with a functional role for GABA signaling in the palate. In addition, *Gad1* gene expression has been detected in several epithelial placodes as well as in non-neural tissues that are developmental signaling centers, suggesting a role for GABA in developmental processes outside of the CNS [18]. In the case of *Viaat*, cleft palate and body wall phenotypes were noted in a previous analysis of a *Viaat* knockout mouse [5]. However, that report did not examine the mechanisms underlying the non-neural phenotypes in the *Viaat* mutants.

In this study we used three independent experimental approaches to show that the non-neural defects in *Gad1* and *Viaat* mutant mice are due to loss of gene function within the CNS during development. We performed a pharmacological rescue experiment to show that the GABA_A_ agonist muscimol can suppress the cleft palate phenotype in *Gad1* and *Viaat* mutant embryos. The ability of muscimol to rescue the cleft palate phenotype of *Gad1* and *Viaat* mutants suggests that multimeric GABA_A_ receptors are downstream of *Gad1* and *Viaat* in palate formation, a pathway that is most consistent with GABA signaling in the CNS. In addition, we used a serum free explant culture system to show that oral explants derived from *Gad1^lacz^* -/- and *Viaat^lacZ^* -/- embryos were competent to undergo normal palate shelf elevation and fusion when removed from their normal oral context. This experiment provided additional evidence that *Gad1* and *Viaat* function are not directly required for palate formation. A third experimental approach was to inactivate *Gad1* specifically in neural precursor cells throughout the CNS. We took advantage of a *Gad1* conditional allele to demonstrate that an early neural precursor specific knockout of *Gad1* was sufficient to cause a cleft palate phenotype in mutant embryos. Our work strongly supports the idea that the cleft palate phenotype in *Gad1* and *Viaat* knockout mice is due to a loss of GABA signaling in the CNS rather than in craniofacial tissues. We interpret our results in the context of other studies that have documented a role for fetal movement in the development of tissues and structures outside of the CNS. We suggest that the phenotypes seen in the *Viaat* and *Gad1* knockout mice belong to a family of fetal defects in non-neural tissues that are caused by a loss of normal fetal movements due to defects in CNS or muscle function during development.

## Materials and Methods

### Mouse strains

All work with mice conformed to the stipulations of the University of Georgia Institutional Animal Care and Use Committee. The University of Georgia animal welfare assurance number is A3437-01 which expires on 11/30/2011. The *Viaat^lacZ^* knockin/knockout allele was generated by gene targeting in ES cells. Genomic clones containing the mouse *Viaat* locus were isolated from a 129/SvEv lambda phage library. A 5.2-kb fragment immediately upstream of the translation start site was used as a 5′ arm and a 2.8-kb fragment starting downstream of the start codon was used as a 3′ arm. A β-galactosidase reporter/neomycin resistance cassette was placed into an NcoI site in the *Viaat* first exon. This NcoI site includes the start codon of the *Viaat* gene [19]. After linearization the targeting vector was electroporated into GSI-1 ES cells (obtained from Genome Systems Inc.). Four targeted ES cell lines were injected into blastocysts and one chimera transmitted the knockout allele to the founder offspring. The *Viaat* knockout mice used in this study had been crossed for 4–10 generations to the C57Bl/6J background. The *Gad1^lacZ^* knockin-knockout mice have been described previously [20]. The strain carrying the floxed conditional allele of the *Gad1* gene was obtained from Dr. Richard Palmiter. This strain has been described previously [6]. The Nestin-Cre strain [21] was obtained from The Jackson Laboratory. Fetal mice were genotyped using yolk sac DNA and adult mice were genotyped using tail DNA as template for PCR.

### Histological analysis

For paraffin sections, embryos were fixed in Bouin's solution at 4°C from 3 hours to overnight depending on the embryonic stage and washed in phosphate buffer (pH 7.3) overnight. Then the embryos were then dehydrated in a graded series of ethanol, equilibrated with xylene, embedded in paraffin and sectioned at a thickness of 10 µm. Sections were stained with Mayer's hematoxylin (Sigma) and eosin solution after dewaxing with xylene, and mounted on slides.

Whole mount lacZ histochemistry was performed on E9.5-E14.5 day old heterozygous *Viaat* embryos. The embryos were fixed in 0.4% paraformaldehyde (PFA), 100 mM sodium phosphate pH 7.3, 2 mM MgCl_2_, 5 mM EGTA for 30–60 minutes and then rinsed in 100 mM sodium phosphate pH 7.3, 2 mM, MgCl_2,_ 0.01% sodium deoxycholate, 0.02% igepal for at least 3 hours and stained in 100 mM sodium phosphate pH 7.3, 2 mM MgCl_2_, 0.01% sodium deoxycholate, 0.02% igepal, 5 mM potassium ferricyanide, 5 mM potassium ferrocyanide, 1 mg/ml X-gal at 37°C. After staining, embryos were washed in PBS and postfixed in 4% PFA/PBS for 1 hour at room temperature.

### Palate Explant Culture

For the explant cultures we developed a new serum free culture system that combined a serum-free *in vitro* mouse embryo culture technique [22] with a previously described method for dissecting the palatal region for in vitro culture [23]. E13.5 Mouse embryos were dissected in Knockout DMEM (Invitrogen). The mandibular region including tongue was removed from the embryo head and the brain tissues were dissected out in parallel at the level of the eyes by using an aseptic scalpel. Also, the remaining tissues from the hindbrain and cerebellum including their covering skin were completely removed with fine forceps. The explants were cultured in a rolling bottle apparatus from BTC Engineering (Cambridge, UK) at 37°C in 3 ml of serum-free media per palate in an atmosphere of 95%O_2_/5% CO_2_. The culture medium was replaced after 24 hr. After culture for 1 or 2 days, explants were processed for paraffin embedding and H&E staining as described above.

### Muscimol rescue experiment

To generate homozygous offspring for the rescue experiments, we intercrossed *Gad1* or *Viaat* heterozygotes. To increase the likelihood that litters were within comparable ranges of gestational age matings were set up at 9pm and the mice were separated at 9am the next day. Muscimol injections were done within the same time window for each set of pregnant dams. This was done to improve the reproducibility of the results between different litters. In the *Gad1*-/- rescue experiment, muscimol was injected into the abdominal cavity of pregnant dams every 24 hours from E13.5 to E15.5. For the *Viaat*-/- rescue experiment, muscimol was injected every 12 hours from E13.5 to E16.0. We used 4 mg/kg of muscimol for each injection. This is a dosage that results in obvious sedation of the mice [24].

### RT-PCR

RT-PCR analysis of mRNA expression was performed as previously described [20]. The primer sequences and expected sizes for each gene are as follows:

*Viaat (578 bp)* 5′-GTCGAGGGAGACATTCATTATCAG-3′, 5′-GTACACAGCAGACTGAACTTGGAC-3′

*Gad1 (302 bp)* 5′-CCTTCGCCTGCAACCTCCTCGAAC-3′, 5′-GCGCAGTTTGCTCCTCCCCGTTCTT-3′

*GAPDH (310 bp)* 5′-GTCTACATGTTCCAGTATGACTCCACTCAC-3′, 5′-CAATCTTGAGTGAGTTGTCATATTTCTCGT-3′

*Gabrb3 (356 bp)* 5′-GAAATGAATGAGGTTGCAGGCAGC-3′, 5′-CAGGCAGGGTAATATTTCACTCAG-3′

*Ucp-1 (593 bp)* 5′-TAGGTATAAAGGTGTCCTAGGGA-3′, 5′-CGCTTGGGTACTGTCCTGG-3′

## Results

### Generation of the *Viaat^lacZ^* and *Gad1^lacZ^* knockin/knockout mice

**Figure 1:**
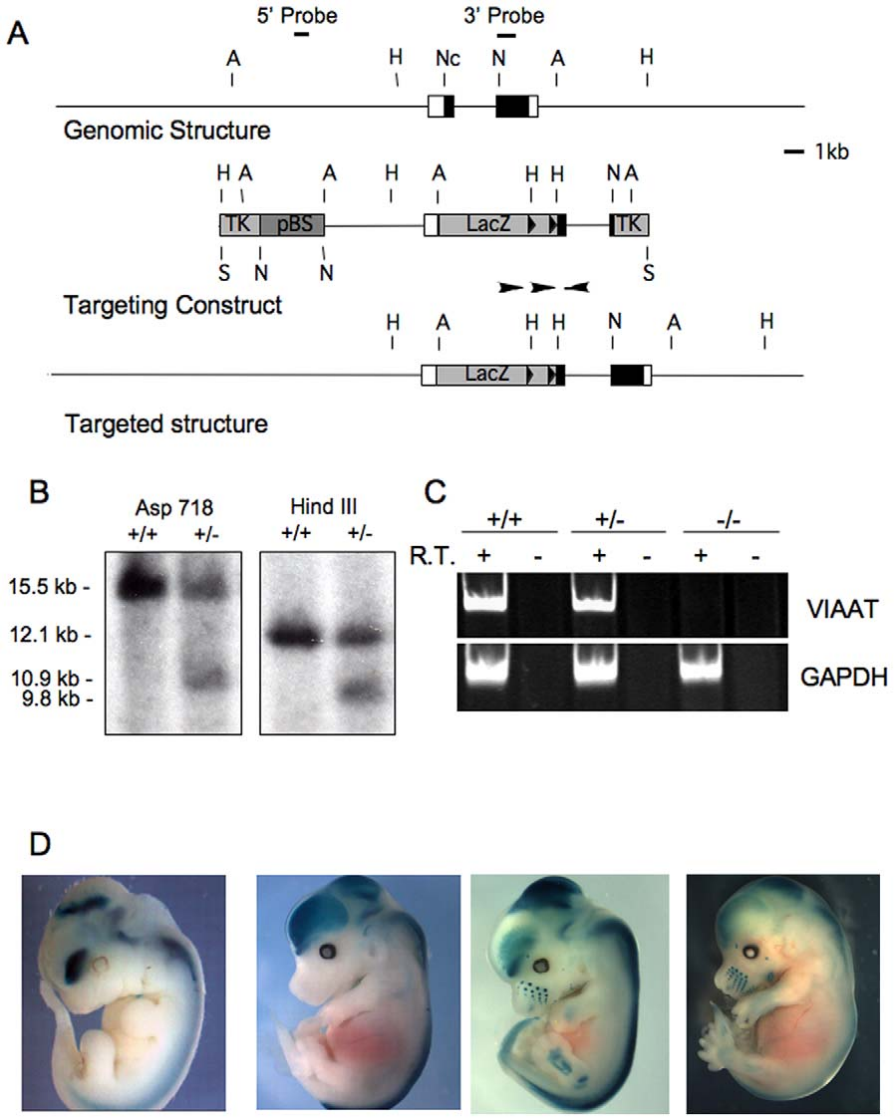
Targeted disruption of the *Viaat* gene in mice. (A) A schematic representation of the *Viaat* wild-type genomic locus (Genomic Structure), the targeting vector (Targeting Construct), and the mutant locus (Targeted structure) are shown. A *LacZ* sequence was inserted into an NcoI site at the *Viaat* start codon. The lox sites that flank the Neo resistance cassette are indicated with two arrowheads in the region immediately 3′ to the *lacZ* sequence. The locations of the 5′ (5′ Probe) and 3′ (3′ Probe) flanking probes used in characterizing the *Viaat* allele are indicated above the map of the wild type *Viaat* locus. The positions of the restriction sites used in the Southern blot analysis of genomic DNA from ES cells are indicated by single letters. Restriction sites are indicated as follows: H (HindIII), Nc (NcoI), A (Asp718) and N (NotI). (B) Southern blot analysis of genomic DNA from a wild type parental ES cell line (+/+) and a targeted ES cell line used to generate the *Viaat^lacZ^* mouse (+/-) are shown. In genomic DNA digested with Asp718 the 3′flanking probe hybridized to a 15.5 kb wild type fragment and a 10.9 kb from the targeted allele (Asp718 panel) and in genomic DNA digested with HindIII this probe hybridized to a 12.1 kb wild type band and a 9.8 kb mutant band (HindIII panel). (C) RT-PCR analysis of cDNA from wild-type (+/+) , heterozygous (+/−), and mutant embryo brain at E16.5. For each genotype RT-PCR was performed on RNA that had been reverse transcribed (+ lanes) and RNA that had not been incubated with reverse transcriptase (−) to control for non-specific amplification. GAPDH primers were used as a positive control. (D) Localized expression of β-galactosidase activity in *Viaat^lacZ^* and Gad1^lacZ^ heterozygotes. The panels from left to right show β-galactosidase activity in E11.5, and E14.5 *Viaat^lacZ^* embryos and in E12.5 and E14.5 *Gad1^lacZ^* embryos.

We generated mice carrying lacZ-tagged knockin/knockout alleles of *Viaat* and *Gad1* for genetic studies and to facilitate the detection of *Viaat* or *Gad1* expressing cells by β-galactosidase histochemistry. The *Viaat^lacZ^* and *Gad1^lacZ^* mice add to the existing mouse resources that express lacZ or fluorescent proteins in GABAergic and/or glycinergic neurons (Table 1). The strains listed in Table 1 carry marker gene knockin alleles or BAC transgenes wherein marker gene expression is likely to recapitulate most or all of the expression pattern of the endogenous gene. Additional transgenic lines have been generated with smaller segments of the regulatory regions of these genes [25], [26], [27]. Previously described knockin/knockout alleles of *Gad1* and *Viaat* were also tagged with marker genes, but expression of the marker from the knockin allele could not be detected [4], [5]. Our *Viaat^lacZ^* knockin allele was generated by inserting a lacZ coding sequence into the first exon of the *Viaat* gene (Figure 1A) [19]. Targeted ES cell clones and mice were identified by Southern blot analysis of genomic DNA (Fig. 1B) and RT-PCR analysis showed that *Viaat* transcripts were undetetctable in the homozygous *Viaat^lacZ^* offpring (Fig. 1C). A similar strategy was used to generate a lacZ tagged allele of *Gad1*. The design of the *Gad1^lacZ^* allele was described previously [20]. In both strains the neo cassette was removed by crossing the founder mice to a germline Cre deleter strain [28]. The studies described here used mice carrying the neo-deleted lacZ knockin/knockout alleles of *Viaat* and *Gad1*.

**Table 1:** Knockin and transgenic mice that express lacZ or EGFP in GABAergic and/or glycinergic neurons.

Mouse strain	Reference
*Gad1^lacZ^* knockout/knockin	This study
*Viaat* ^lacZ^ knockout/knockin	This study
*Gad1*-EGFP knockout/knockin	[17]
*Gad1*-EGFP BAC transgenic	[76]
Gad2-EGFP BAC transgenic	www.gensat.org
*Viaat* (Slc32a1)-EGFP BAC transgenic	www.gensat.org
Glyt2 (Slc6a5)-EGFP BAC transgenic	www.gensat.org; [77]

To validate the expression of the lacZ marker from the *Viaat^lacZ^* and *Gad1^lacZ^* knockin alleles, we performed an initial analysis of β-galactosidase expression in the knockin mice. Whole mount and section Xgal histochemistry revealed expression patterns in both mouse strains that mirrored our previously published *in situ* hybridization results (Fig. 1D)[18], [19].

### *Viaat* is required for normal palate and ventral body wall development

**Figure 2:**
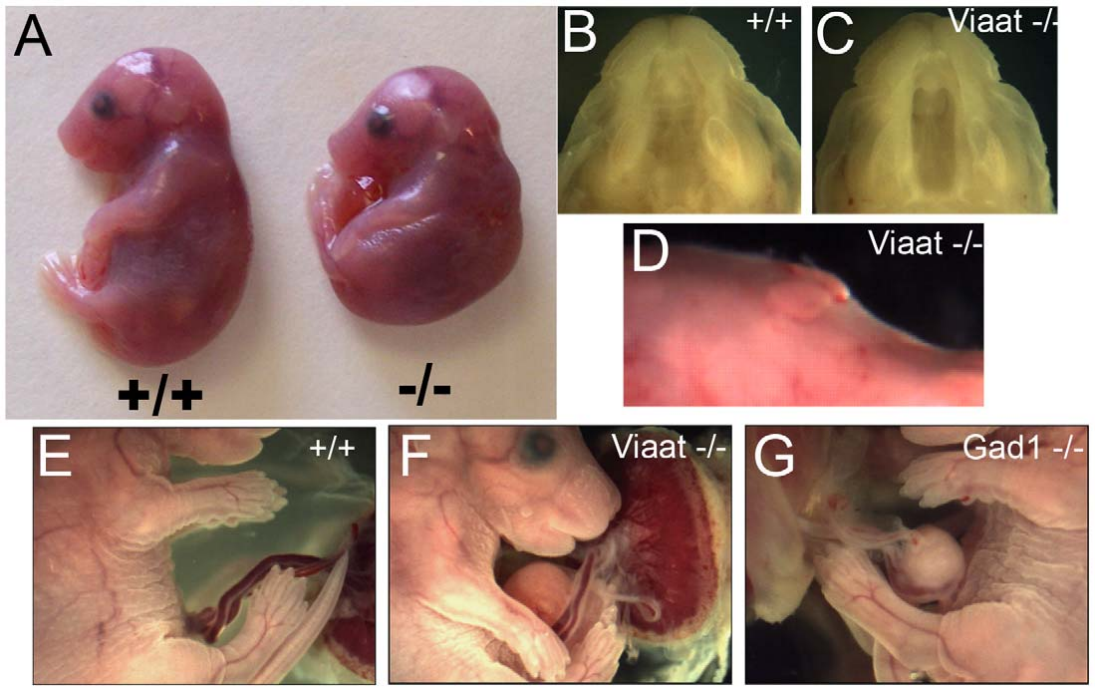
*Viaat* mutants exhibit cleft palate and umbilical hernia. (A) Gross morphology of a *Viaat^lacZ^* (−/−) mutant at E18.5 with hunched posture as compared to a wild type E18.5 littermate (+/+). (B, C) Examination of E17.5 embryos showed that all of the *Viaat^lacZ^* mutants exhibited a cleft palate (C) as compared to wild type (B). (D) *Viaat* mutants have small subcutaneous bumps on the dorsal side of cervical region. (E, F) Compared to wild type (E) nearly all of the *Viaat^lacZ^* mice exhibited an umbilical hernia (F). (G) *Gad1^lacZ^* mutant E17.5 mice also displayed a similar hernia phenotype.

To define the spectrum of mutant phenotypes in the *Viaat^lacZ^* knockout mouse we examined newborn progeny from intercrosses of *Viaat^lacZ^* heterozygous mice. The *Viaat^lacZ^*-/- progeny were immediately identifiable due to their lack of movement, hunched posture, and failure to breathe (Fig. 2A). All *Viaat^lacZ^* homozygous offspring died at or immediately prior to birth. Examination of the pups showed that the *Viaat^lacZ^* -/- newborns exhibited a cleft secondary palate, umbilical hernia and small bumps under the skin on the dorsal side of the cervical region (Fig. 2B-2F). Histological examination of paraffin sections showed that none of the *Viaat^lacZ^* -/- newborns examined had inflated their lungs at birth indicating an early fundamental defect in respiratory function (data not shown).

**Figure 3:**
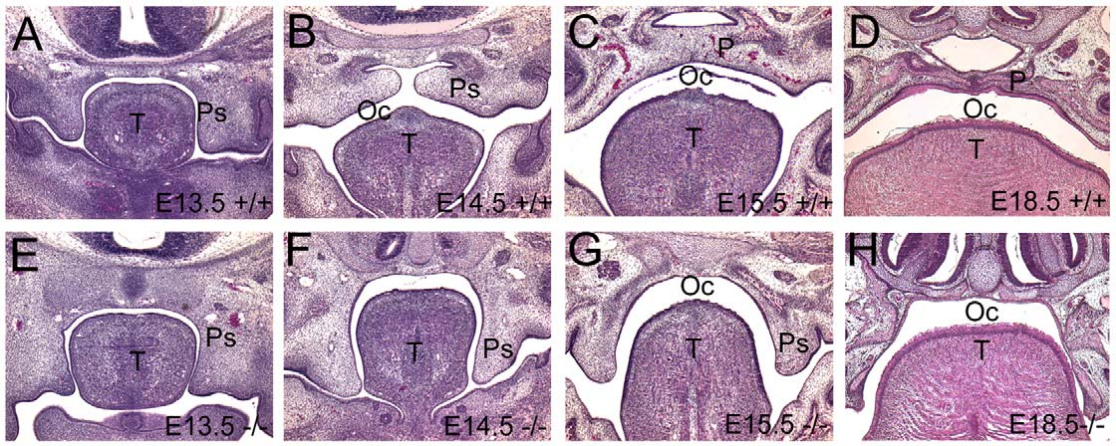
*Viaat* mutants exhibit delays in palate shelf elevation. (A-D) H&E stained coronal sections showing normal palatogenesis in wild type E13.5, E14.5, E15.5 and E18.5 mice. (E-H) Coronal sections of *Viaat^lacZ^* homozygous mutants at E13.5, E14.5, E15.5 and E18.5, showing a failure of palate shelf elevation in the mutants. P, palate; PS, palatal shelf; OC, oral cavity; T, tongue.

We compared the *Viaat^lacZ^* -/- newborn phenotype to that of *Gad1^lacZ^*-/- newborns. Nearly all (98%) of the newborn *Viaat^lacZ^* homozygotes exhibited umbilical hernias (Fig. 2F, G). Examination of the *Gad1^lacZ^* homozygotes revealed a similar, but less frequent (85%) umbilical hernia phenotype that had not been previously reported. Histological sections of *Viaat^lacZ^* homozygotes revealed that this defect was indeed an umbilical hernia, not an ompahalocele as previously reported for the *Viaat* homozygous mice [5]. Omphalocele is a severe body wall defect that results in an abnormally large umbilical ring usually exposing the gut and liver [29], [30]. In contrast, an umbilical hernia is caused by a failure to retract the umbilical hernia that occurs normally during development [29], [30]. These phenotypes suggest that *Gad1* and *Viaat* functions are not necessary for body wall formation *per se* but are instead required for the retraction of the umbilical hernia during development. In both genotypes, palate shelf elevation fails to occur at E14.5 and all *Gad1* and *Viaat* mutants were born with cleft secondary palates (Fig. 3A-H and data not shown).

We also examined the unusual “bumps” on the dorsal cervical region of the *Viaat^lacZ^* -/- mice (Fig. 2D). These “bumps” were not present in *Gad1* mutant newborns. Our analysis indicates that they are clumps of displaced brown fat since they resemble mouse brown fat in H&E stained paraffin sections and RNA extracted from the bumps contained very high levels of Ucp1 mRNA (data not shown). Ucp1 is a specific marker of brown fat [31], [32]. In a newborn mouse the major deposit of brown fat is found in the intrascapular region, with additional deposits in various locations in the cervical region [33]. Presumably, the hunched posture and the apparent muscular paralysis exhibited by the *Viaat^lacZ^* homozygotes leads to the displacement of these fat deposits to this abnormal location.

### *Viaat* is not expressed in the developing palate

**Figure 4:**
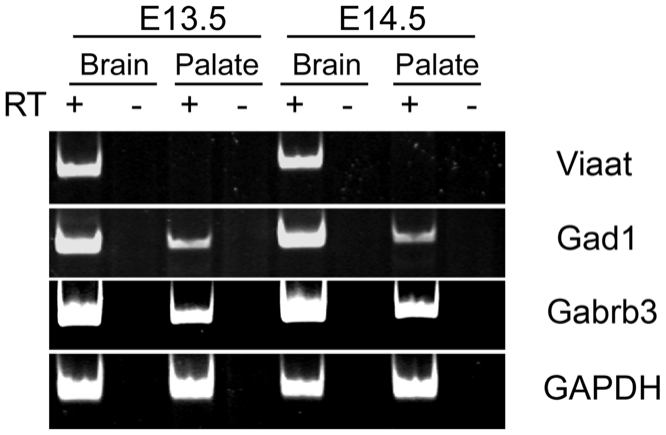
The developing palatal shelves do not express *Viaat* transcripts. RNA from dissected E13.5 and E14.5 brain tissue (Brain) or E13.5 and E14.5 dissected palate shelves (Palate) was analyzed by RT-PCR using primers corresponding to the *Viaat*, *Gad1*, *Gabrb3* and *Gapdh* coding sequences. For each tissue and stage RT-PCR was performed with cDNA (+) or with RNA that had not been reverse transcribed (−) to control for non-specific amplification. *Gapdh* was used as a positive control.

Previous studies have suggested the possibility that GABA may be functioning as a signaling molecule in non-neuronal tissues during development [10], [18], [34]. Previous gene expression studies have shown that *Gabrb3* receptor subunit and *Gad1* mRNAs are expressed in the developing palate and teeth as well as in a variety of other non-neuronal cell types [10], [15], [16], [18], [35]. If *Viaat* function is required in the palate or craniofacial structures it should be expressed in these tissues. To determine whether *Viaat* mRNA is expressed in the developing palate, we examined the expression of *Gabrb3*, *Gad1* and *Viaat* in mRNA from dissected palatal shelves from E13.5 and E14.5 day old mice. Using RT-PCR we easily detected *Gad1* and *Gabrb3* transcripts but could not detect *Viaat* mRNA in this tissue (Fig. 4). These results indicate that *Viaat* is not expressed in the palatal shelves at the time of palate shelf elevation and fusion. The lack of *Viaat* expression in the palate suggests that its function is not required in the palate for its normal development.

### Palate explants from *Gad1* and *Viaat* mutants can undergo shelf elevation and fusion

**Figure 5:**
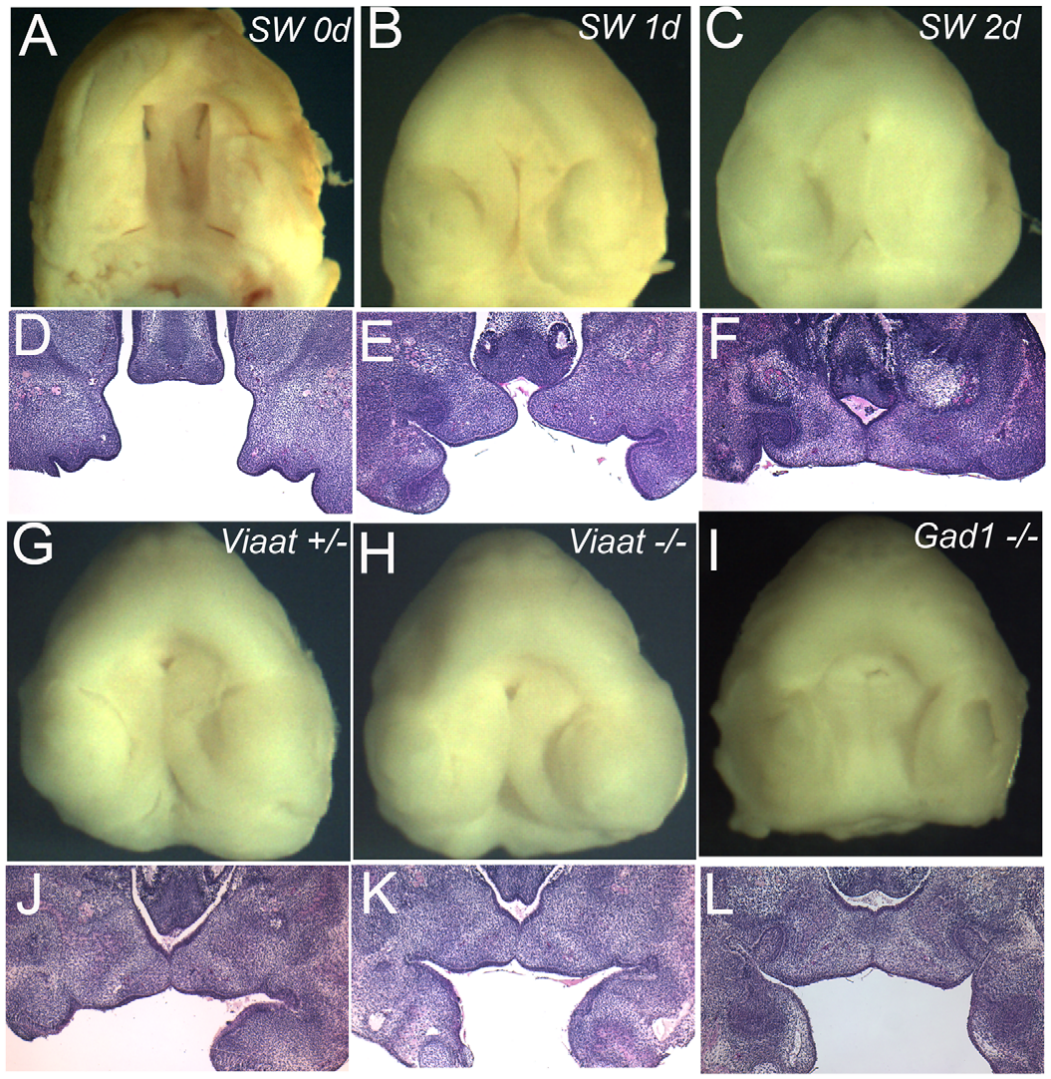
*In vitro* fetal palate explant culture. (A-F) Wild-type palates from Swiss Webster (SW) embryos at E13.5 were dissected and cultured for 2 days. (A-C) View of the oral surface of explants prior to culture (A) and after 1 day (B) or 2 days (C) of culture. (D-F) sections through the palate explants in shown in panels A-C. (G-I) Normal palatogenesis of the *Viaat* heterozygous (+/−) (G) and homozygous (−/−) (H) explants after 2 days in culture. Palate explants from the *Gad1* mutant embryo at E13.5 also developed normally during culture for 2 days (I). (J-L) Sections of the palate explants shown in panels G-I.

A previous study showed that oral explants made from *Gad1* knockout mice could undergo palatal shelf elevation in embryo culture [13]. In this previous study, the authors also performed exo-utero surgical manipulations that suggested that removal of the tongue was sufficient to allow normal palatogenesis in the *Gad1* knockout embryos. Building on these previous results, we performed a series of explant experiments to test whether there is a requirement for GABA signaling within the developing palate shelves for normal shelf elevation and fusion in the *Viaat^lacZ^* -/- embryos and control littermates. This experiment is a simple way to test whether the mutant palate is capable of undergoing normal morphogenesis when removed from the context of the embryo. We developed an explant culture system that used a previously described dissection protocol to generate explants from the maxillary region of the E13.5 mouse embryos [23]. Explants dissected using this protocol were also used in the previous analysis of palate development in the *Gad1* mutant explants [13]. We cultured the explants in a serum free and chemically defined medium that we had previously developed for mouse embryo culture [22]. In this system wild type explants underwent palate shelf formation, growth, elevation and fusion thereby recapitulating the normal events in palatogenesis (Fig. 5A-F). Explants from *Viaat* ^lacZ^-/- and *Gad1^lacZ^*-/- embryos also underwent shelf elevation and fusion in this explant culture system (Fig. 5G-L). The absence of cleft palate in the *Viaat* and *Gad1* mutant explants after two days reproduces the previous results seen with the *Gad1* -/- explants and shows that palates from the *Viaat^lacZ^* mutants are also able to undergo palatogenesis in this system. In total we tested 5 *Viaat^lacZ^* mutant explants from 3 different litters and 3 *Gad1^lacZ^* mutant explants derived from 3 different litters. Wild type and heterozygous explants from these litters served as controls. In all cases the *Viaat^lacZ^* and *Gad1^lacZ^* mutant explants underwent palatal shelf outgrowth and elevation in culture. In both the mutants and controls all of the palatal shelves fused to some extent with most of the explants exhibiting nearly complete fusion. There was no difference in the overall extent of fusion observed in control and mutant explants. Normal palate shelf elevation in the explants is consistent with there not being a requirement for GABA signaling within the palate for normal palatogenesis. However, on their own the explant experiments cannot completely exclude a possible role for GABA in the palate shelf itself.

### The GABA agonist muscimol suppresses the cleft palate phenotype in the *Viaat* and *Gad1* homozygous embryos

The presence of a cleft palate phenotype in *Gad1*, *Viaat* and *Gabrb3* mutant mice suggests that the three genes function in a GABA signaling pathway required for palatogenesis. If this signaling pathway is dependent on functional GABA_A_ receptors, as suggested by the phenotype of the *Gabrb3* knockout mouse, then specific pharmacological agonists of GABA_A_ receptors might suppress or “rescue” the phenotypes seen in *Gad1^lacZ^* -/- and *Viaat^lacZ^* -/- embryos. We tested whether the specific GABA_A_ agonist muscimol can suppress the non-neural phenotypes of the *Gad1* and *Viaat* homozygous embryos. We chose to use muscimol as an agonist because it is a potent GABA_A_ receptor agonist that crosses the placental barrier and it binds to the same binding pocket on pentameric GABA_A_ receptors as GABA [36], [37]. Since muscimol binds to a pocket formed by an alpha and a beta GABA_A_ subunit in a multimeric GABA_A_ receptor, any rescue of the *Gad1^lacZ^* or *Viaat^lacZ^* mutant phenotypes by muscimol would suggest that multimeric GABA_A_ receptors were the downstream target of *Gad1* or *Viaat* function [36], [38], [39], [40].

We injected muscimol into pregnant *Viaat^lacZ^* +/- or *Gad1^lacZ^* +/- dams that had been mated with *Viaat^lacZ^* +/- or *Gad1^lacZ^* +/- males respectively. Multiple muscimol injections from E13.5-E16 days were performed and the resulting offspring were removed at E17.5 days for examination. IP injection of muscimol into the pregnant dams did suppress the development of cleft palate in *Viaat^lacZ^* -/- and *Gad1^lacZ^*-/- mice but did so to different extents in the two genotypes. Muscimol injection suppressed the formation of cleft palate in about 20% of the *Viaat^lacZ^* homozygous mutant embryos and in about half of the *Gad1* homozygous mutant embryos (Table 2). Since the cleft palate phenotype is fully penetrant in *Viaat* and *Gad1* homozygous fetuses and newborns, the rescue of the cleft palate phenotype in mutant embryos exposed to muscimol is quite clear. Histological analysis of rescued *Viaat^lacZ^* -/- E17.5 embryos showed that palate shelf elevation and fusion had occurred in all of the rescued homozygotes while in the case of the *Gad1^lacZ^* -/- rescued embryos palate shelf elevation and fusion had occurred in 2 of the 7 rescued homozgyotes while shelf elevation without fusion had occurred in the other 5 rescued *Gad1^lacZ^*-/- fetuses.

**Table 2:** Frequency of rescue of cleft palate by muscimol in *Gad1*-/- and *Viaat*-/- mice.

Frequency of Cleft Palate			
Genotype	PBS^a^	Muscimol (Viaat)	Muscimol (Gad1)
+/+	0/19	1/21	1/10
+/−	1/46	0/34	1/31
−/−	19/19	16/20	7/14

The frequency of cleft palate is expressed as the number of E17.5 day mice exhibiting a cleft palate over the total number examined for that injection condition.

The frequency of cleft palate in *Gad1*-/- E17.5 mice is 100%. In the case of the *Viaat* mutant mice we have examined over 200 newborn or late gestation embryos and the cleft palate phenotype is present in 100% of the offspring.

a PBS injected controls were *Viaat* +/− dams from *Viaat^lacZ^* heterozygote intercrosses.

### *Gad1* function in the CNS is required for normal palate formation

To perform a genetic test of the requirement for *Gad1* function in the CNS for normal development we examined the phenotype of mice with a CNS-specific knockout of *Gad1*. Because palate shelf elevation and fusion occur between E13.5 and E14.5 we chose to inactivate *Gad1* throughout the CNS in stem cells and progenitors well before these events in palate morphogenesis. To inactivate *Gad1* in the early CNS we used a well-characterized transgenic line (NesCre) that expresses Cre under the control of the Nestin regulatory sequences [21]. The NesCre strain expresses Cre exclusively in neural precursors starting at E9.5 days and by E11.5 days the expression of Cre activity is widely and uniformly distributed throughout the nervous system [21], [41], [42], [43]. This strain is known to lack any detectable cre expression in the craniofacial region [44]. The floxed allele of *Gad1* used in our experiments has been described previously [6], [45].

**Figure 6:**
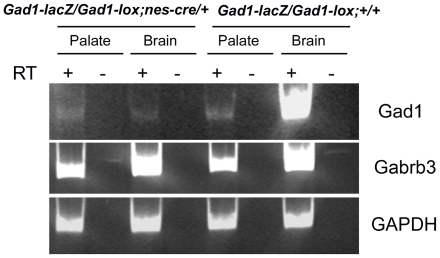
Specific inactivation of *Gad1* in the CNS of *Gad1^flox^/Gad1^lacZ^* NesCre E14.5 day old embryos. RT-PCR analysis of RNA extracted from dissected palate shelves (Palate) or brain (Brain). The genotype of the tissue is indicated above. For each sample PCR amplification was performed with cDNA (+ lanes) as well as RNA that had not been reverse transcribed (- lanes).

To generate offspring with a CNS specific inactivation of *Gad1* we crossed *Gad1^lacZ^/+*; *NesCre* mice to *Gad1^flox^/Gad1^flox^* mice. To confirm that *Gad1* had been inactivated specifically in the CNS of the *Gad^flox^/Gad^lacZ^* ; *NesCre/+* offspring from this cross we used RT-PCR to measure *Gad1* mRNA levels in RNA isolated from dissected palatal shelves and brain from E14.5 embryos. The RT-PCR analysis showed that the amount of *Gad1* mRNA in the palatal shelves of the CNS specific *Gad1* knockout embryos (*Gad^flox^/Gad^lacZ^*; *NesCre*) was equivalent to controls, while *Gad1* mRNA levels in the CNS were greatly reduced as compared to the control embryos (Fig. 6). Examination of *Gad^flox^/Gad1^lacZ^*; NesCre embryos at E17.5 showed that the cleft palate and the body wall phenotypes were present in the CNS-specific knockout. In total, we found a complete cleft palate in 9 out of the 11 *Gad^flox^/Gad1^lacZ^* NesCre E17.5 embryos that we examined. This cleft palate phenotype was identical to that seen in Gad1 mutant fetuses and newborns. We also found that 6 of the CNS specific E17.5 *Gad^flox^/Gad^lacZ^*; *NesCre/+* exhibited an umbilical hernia. This genetic test demonstrated that *Gad1* function was required within the CNS for normal development of the non-neural tissues affected in the *Gad1* knockout.

## Discussion

There have been numerous observations published over the last 25 years that implicate GABA signaling in the normal development of non-neural tissues [2], [3], [4], [10], [11], [14], [18], [34], [46], [47], [48]. A role for GABA signaling in non-neural tissues is plausible given the fact that GABA and other neurotransmitters have well-documented roles in the development of tissues and cell types outside of the CNS. A striking and prominent example of this was a recent report demonstrating that activation of GABA_A_ receptors alters embryonic stem cell proliferation in cell culture as well as cell proliferation in mouse blastocyst stage embryos [48]. These cell types exist prior to the earliest formation of the CNS, clearly demonstrating a role for GABA signaling in a completely non-neuronal context. In addition, serotonin, dopamine, acetylcholine, epinephrine and norepinephrine each play important roles in the development, maintenance and/or function of organs and tissues that are outside of the CNS [49], [50], [51], [52], [53], [54], [55], [56]. In these cases receptors for the transmitters are expressed on the non-neural cell types that respond to them [50], [52], [57], [58]. Viewed from this perspective the idea that GABA may be a signaling molecule in developing cell types outside of the nervous system is a reasonable hypothesis. In this study, we designed a set of experiments to test the hypothesis that GABA is required in non-neural cell types for the normal development and morphogenesis of the palate and retraction of the umbilical hernia.

In one set of experiments we found that muscimol was able to suppress the cleft palate phenotype in the *Gad1* and *Viaat* mutant mice. The ability of muscimol to suppress or rescue the non-neural phenotypes in the *Gad1* and *Viaat* knockouts suggested that tonic activation of multimeric GABA_A_ receptors was sufficient to provide normal function. The ability of muscimol to rescue suggests that it can substitute for the signal that is normally generated via the activity of the *Gad1* or *Viaat* genes. The simplest explanation for the rescue is that the muscimol was acting on multimeric GABA_A_ receptors in the developing CNS. On the other hand, if muscimol was acting on non-neural cells then GABA_A_ receptor subunit transcripts would be expressed in these cells, presumably in cells adjacent to *Gad1* expressing cells. Although we detected *Gabrb3* transcripts in palate RNA by RT-PCR we have not been able to detect localized expression of the *Gabrb3* mRNA by *in situ* hybridization. In addition, surveys of gene expression in the developing mouse palate have not detected the expression of any GABA_A_ receptor subunits besides the *Gabrb3* subunit [16], [35]. Therefore, the rescue of the cleft palate phenotype in the *Viaat* and *Gad* mutants by muscimol is consistent with the requirement for GABA signaling within the CNS for normal palate development. However, this point requires additional verification and support via a systematic analysis of GABA receptor expression within the palatal shelves to confirm that muscimol rescues the *Gad1^lacZ^* and *Viaat^lacZ^* mutant phenotypes by acting on receptors in the CNS.

The results of our explant culture experiments and the CNS specific knockout of *Gad1* are also consistent with a requirement for GABA signaling in the CNS for normal palate and body wall development. Our explant experiments repeated the results previously obtained with *Gad1* mutant explants [13]and demonstrated that *Viaat* mutant oral explants can also undergo palate shelf elevation and fusion in culture. The ability of the *Viaat* mutant oral explants to undergo palatogenesis suggested that the cleft palate phenotype of *Viaat* mutants is not due to a defect within the palate shelves themselves. Consistent with these observations our CNS specific knockout of *Gad1* in neural precursor cells showed the loss of *Gad1* function specifically within the CNS was sufficient to cause a cleft palate and body wall phenotype. This genetic result confirms that *Gad1* function and GABA signaling within the CNS is necessary for palate and body wall formation.

In contrast, some previously published studies support a developmental function for GABA signaling outside of the CNS. Most of these results came from genetic studies of the gene encoding the β3 subunit of the GABA_A_ receptor (*Gabrb3*). Inactivation of *Gabrb3* in mice led to the development of cleft palate in a proportion of the homozygous offspring [1], [2], [11]. In one study the expression of *Gabrb3* was driven by the neuron specific enolase (NSE) regulatory sequences in a genetic background that lacked *Gabrb3* [10]. The authors found that the development of cleft palate was not suppressed in these transgenic mice. This result was interpreted as evidence that *Gabrb3* function is required in the palate or other non-neural tissues for normal palate development [10]. However, no detailed characterization of the NSE-*Gabrb3* temporal and spatial expression pattern in the transgenic embryos was provided. This is important since NSE transgenes can be subject to position effects resulting in very large differences in the spatial expression pattern of expression within the CNS between different transgenic lines [59], [60]. In addition the expression of NSE transgenes is minimal at E13.5–14.5 during the time of palate elevation and fusion [61]. In fact, NSE sequences are used to drive gene expression in postmitotic neurons during later stages of development [62]. Without additional information it is not possible to know whether *Gabrb3* was being expressed early enough in the NSE-*Gabrb3* transgenic lines tested in this previous study.

A second set of observations supporting a developmental function for GABA in non-neural tissues comes from studies of a neuron-specific knockout of the *Gabrb3* gene [11]. In these experiments, a floxed allele of the *Gabrb3* gene was inactivated specifically in neurons by crossing to a synapsin-Cre transgenic line [11], [63], [64]. The authors observed that the inactivation of *Gabrb3* specifically in neurons resulted in normal palate development in the mice. Although the synapsin-Cre (SynCre) line is well characterized and has been used to generate neuron specific gene inactivation, the timing of expression may not be ideal for assessing *Gabrb3* function in palate formation. The SynCre transgene is specifically expressed in postmitotic neurons and is not widely expressed in the CNS until E13.5 days [63], [64], [65]. It is likely that in the SynCre *Gabrb3^flox^* mice the Cre may inactivate the conditional Gabrb3 allele too late in development to interfere with palate elevation and fusion which occur during E13.5-E14.5. This is a particular concern since the authors were relying on the inactivation of two *Gabrb3^flox^* alleles in the offspring. Unfortunately, the authors did not monitor *Gabrb3* transcript levels in the CNS and palate of the SynCre *Gabrb3^flox^*/*Gabrb3^flox^* mice or the extent of Cre mediated recombination of the *Gabrb3* ***^flox^*** allele in these experiments [11].

### GABA signaling in the CNS is required for fetal movement and developmental processes that depend on fetal movement

Fetal akinesia during human or rodent development has been shown to cause defective development of several non-neural tissues and structures in the fetus. For example, it has been shown that normal lung development requires spontaneous fetal breathing movements in humans and mice [66], [67], [68]. In humans developmental defects caused by fetal akinesia include multiple pterygium syndrome (OMIM #265000 and #253290) and the fetal akinesia deformation sequence (FADS; OMIM #208150). The phenotypes associated with these syndromes include pulmonary hypoplasia, craniofacial defects, joint contractures, limb defects, skeletal defects, webbing of the skin (pterygia) and cardiac defects. Studies have suggested that in some cases FADS is found in infants born to mothers who have myasthenia gravis, an autoimmune disorder that affects acetylcholine receptors in the neuromuscular junction [69], [70]. Recent work has tied several human FADS and multiple pterygium syndrome cases to mutations in nicotinic acetylcholine receptors as well as mutations in RAPSN a protein that is associated with acetylcholine receptors [71], [72], [73]. This demonstrates that defects in neuromuscular junction components can lead to non-neural developmental defects. These reports provide genetic evidence that mutations in genes required for neuromuscular function can cause a spectrum of non-neural defects.

Within this context, the craniofacial and body wall phenotypes found in *Gad1* and *Viaat* knockouts can be understood as additional examples of non-neural phenotypes caused by changes in fetal CNS function that in turn lead to changes in fetal muscle tone or fetal movements. Previous work has shown that fetal movements during palate formation are impaired in the *Gad1* mutant mice [12]. Although the phenotypes are secondary defects caused by the primary defect of disrupted GABA signaling in the CNS, they are fully penetrant developmental defects that are similar to some of the most common developmental defects seen in humans. To develop a comprehensive understanding of the origins of defects in human development it is important to understand the diverse mechanisms, both primary and secondary, that can cause or increase the likelihood of such defects. We feel that the phenotypes seen in the *Gad1* and *Viaat* homozygous mice are part of a larger spectrum of developmental defects and disorders that are caused by impaired or absent fetal movement. Our work suggests that disruptions of GABA signaling during development could interfere with any one of several developmental processes that depend on fetal movement.

In this context is intriguing that there are correlations between the timing of initial fetal movements in mice or humans and the initiation of palatogenesis. In mice, palate elevation and fusion occur at the same time as the first fetal movements are detected [12]. In humans fetal movements start at 7–8 weeks, with breathing and hiccup like movements initiating at 8–9 weeks [74]. This coincides with the time of palate shelf elevation during the 8th week of human development ([75]). The similarity in timing of human palate elevation and the initiation of fetal movements suggests that alterations in GABA signaling could interfere with human palate development or the development of any other structure or tissue that develops after the initiation of fetal movements. So far, genetic studies have detected weak associations between *Gad1* or *Gabrb3* alleles and human oral clefts [7], [8], [9]. Perhaps the weak association of *Gad1* and *Gabrb3* with oral clefts is due to the fundamental requirement for normal GABA neurotransmission for late fetal or neonatal viability. This would eliminate individuals who are homozygous for null or strong loss of function alleles of *Gabrb3* or *Gad1* from the populations sampled for these studies.

In conclusion, our work provides multiple lines of evidence that the non-neural developmental defects in *Gad1* and *Viaat* mutant mice are due to a requirement for GABA signaling in the CNS during mouse fetal development. The non-neural defects in these mice are most likely due to defects in fetal movement caused by CNS dysfunction and appear to be part of a spectrum of defects caused by abnormalities in fetal movements. Our results help to clarify the mechanism leading to cleft palate in the *Gad1* and *Viaat* knockout mice and suggest that defects in fetal movement caused by alterations in fetal neuronal GABA signaling may lead to similar defects in humans.
